# NSAID use and clinical outcomes in COVID-19 patients: a 38-center retrospective cohort study

**DOI:** 10.1186/s12985-022-01813-2

**Published:** 2022-05-15

**Authors:** Justin T. Reese, Ben Coleman, Lauren Chan, Hannah Blau, Tiffany J. Callahan, Luca Cappelletti, Tommaso Fontana, Katie R. Bradwell, Nomi L. Harris, Elena Casiraghi, Giorgio Valentini, Guy Karlebach, Rachel Deer, Julie A. McMurry, Melissa A. Haendel, Christopher G. Chute, Emily Pfaff, Richard Moffitt, Heidi Spratt, Jasvinder A. Singh, Christopher J. Mungall, Andrew E. Williams, Peter N. Robinson

**Affiliations:** 1 Environmental Genomics and Systems Biology Division, Lawrence Berkeley National Laboratory, Berkeley, CA USA; 2 The Jackson Laboratory for Genomic Medicine, Farmington, CT USA; 3 Translational and Integrative Sciences Center, Oregon State University, Corvallis, OR USA; 4 Computational Bioscience, University of Colorado Anschutz Medical Campus, Boulder, CO USA; 5 Center for Health AI, University of Colorado Anschutz Medical Campus, Aurora, CO USA; 6 AnacletoLab, Dipartimento Di Informatica, Università Degli Studi Di Milano, Milan, Italy; 7 Palantir Technologies, Denver, CO USA; 8 CINI, National Laboratory in Artificial Intelligence and Intelligent Systems—AIIS, Rome, Italy; 9 University of Texas Medical Branch, Galveston, TX USA; 10 Schools of Medicine, Public Health, and Nursing, Johns Hopkins University, Baltimore, MD USA; 11 North Carolina Translational and Clinical Sciences Institute (NC TraCS), University of North Carolina at Chapel Hill, Chapel Hill, NC USA; 12 Department of Biomedical Informatics, Stony Brook University, Stony Brook, NY USA; 13 University of Alabama at Birmingham, Birmingham, AL USA; 14 Medicine Service, VA Medical Center, Birmingham, AL USA; 15 Tufts Medical Center Clinical and Translational Science Institute, Tufts Medical Center, Boston, MA USA; 16 Institute for Clinical Research and Health Policy Studies, Tufts University School of Medicine, Boston, USA; 17 OHDSI Center at the Roux Institute, Northeastern University, Boston, USA; 18 Institute for Systems Genomics, University of Connecticut, Farmington, CT USA

**Keywords:** COVID-19, NSAIDs, Cyclooxygenase inhibitors, Observational study

## Abstract

**Background:**

Non-steroidal anti-inflammatory drugs (NSAIDs) are commonly used to reduce pain, fever, and inflammation but have been associated with complications in community-acquired pneumonia. Observations shortly after the start of the COVID-19 pandemic in 2020 suggested that ibuprofen was associated with an increased risk of adverse events in COVID-19 patients, but subsequent observational studies failed to demonstrate increased risk and in one case showed reduced risk associated with NSAID use.

**Methods:**

A 38-center retrospective cohort study was performed that leveraged the harmonized, high-granularity electronic health record data of the National COVID Cohort Collaborative. A propensity-matched cohort of 19,746 COVID-19 inpatients was constructed by matching cases (treated with NSAIDs at the time of admission) and 19,746 controls (not treated) from 857,061 patients with COVID-19 available for analysis. The primary outcome of interest was COVID-19 severity in hospitalized patients, which was classified as: moderate, severe, or mortality/hospice. Secondary outcomes were acute kidney injury (AKI), extracorporeal membrane oxygenation (ECMO), invasive ventilation, and all-cause mortality at any time following COVID-19 diagnosis.

**Results:**

Logistic regression showed that NSAID use was not associated with increased COVID-19 severity (OR: 0.57 95% CI: 0.53–0.61). Analysis of secondary outcomes using logistic regression showed that NSAID use was not associated with increased risk of all-cause mortality (OR 0.51 95% CI: 0.47–0.56), invasive ventilation (OR: 0.59 95% CI: 0.55–0.64), AKI (OR: 0.67 95% CI: 0.63–0.72), or ECMO (OR: 0.51 95% CI: 0.36–0.7). In contrast, the odds ratios indicate reduced risk of these outcomes, but our quantitative bias analysis showed E-values of between 1.9 and 3.3 for these associations, indicating that comparatively weak or moderate confounder associations could explain away the observed associations.

**Conclusions:**

Study interpretation is limited by the observational design. Recording of NSAID use may have been incomplete. Our study demonstrates that NSAID use is not associated with increased COVID-19 severity, all-cause mortality, invasive ventilation, AKI, or ECMO in COVID-19 inpatients. A conservative interpretation in light of the quantitative bias analysis is that there is no evidence that NSAID use is associated with risk of increased severity or the other measured outcomes. Our results confirm and extend analogous findings in previous observational studies using a large cohort of patients drawn from 38 centers in a nationally representative multicenter database.

**Supplementary Information:**

The online version contains supplementary material available at 10.1186/s12985-022-01813-2.

## Background

As of November 2021, severe acute respiratory syndrome associated with coronavirus-2 (SARS-CoV-2) has infected more than 248 million people and caused more than 5 million deaths worldwide [1]. SARS-CoV-2 is the cause of the coronavirus disease of 2019 (COVID-19), a condition characterized by pneumonia, hyperinflammation, hypoxemic respiratory failure, a prothrombotic state, cardiac dysfunction, substantial mortality, and persistent morbidity in some survivors [2, 3].

Non-steroidal anti-inflammatory drugs (NSAIDs) are a large and heterogeneous class of medications defined by their ability to inhibit cyclooxygenase (COX), an enzyme that catalyzes the conversion of arachidonic acid to prostaglandins. Due to their widespread use, NSAIDs are common causes of serious adverse events that frequently necessitate hospitalization [4].

NSAIDs have numerous potentially deleterious effects on immune function [5, 6] and may also mask warning signs of severe infection such as fever and pain during the course of community-acquired pneumonia [7]. NSAID exposure in the early stage of community-acquired pneumonia has been associated with a delayed diagnosis and more severe clinical course [8, 9], but the quality of available research has been called into doubt and recent studies have failed to reproduce the proposed association [10, 11]. In a mouse model of COVID-19, NSAID treatment reduced both the antibody and proinflammatory cytokine response to SARS-CoV-2 infection. However, the timing of NSAID treatment may be relevant. It is conceivable that early NSAID treatment may negatively impact the initiation of antiviral immune responses while later NSAID treatment could be beneficial by suppressing immune-driven pathology such as cytokine storm [12, 13], but evidence for this supposition is lacking.

A study published early in the course of the pandemic suggested that ibuprofen use was associated with more severe COVID-19 outcomes [8, 14]. However, several subsequent studies failed to demonstrate a significant association between NSAID use and adverse outcomes in COVID-19 patients [10, 15–26]. A prospective, multicenter cohort study on 78,674 hospitalized COVID-19 patients across 255 health-care facilities in England, Scotland, and Wales showed that NSAID use was not associated with worse in-hospital mortality, critical care admission, requirement for invasive ventilation, requirement for non-invasive ventilation, requirement for oxygen, or occurrence of acute kidney injury (AKI) [27]. Finally, a study on OpenSAFELY, an English data analytics platform, showed no evidence of a difference in the risk of COVID-19-related death associated with current use of NSAIDs among 2,463,707 individuals, 536,423 of whom had recorded NSAID use. The same study demonstrated a lower risk of COVID-19-related death was associated with current use of NSAIDs in a cohort of 1,708,781 individuals with rheumatoid arthritis/osteoarthritis, 175,495 of whom had recorded NSAID use [28].

Theoretical considerations suggest a potentially deleterious effect of NSAIDs especially early in the clinical course of COVID-19. The widespread use of NSAIDs coupled with the difficulty of performing a randomized clinical trial on over-the-counter medications make it essential to assess the safety of this class of medication in different settings.

Here, we leverage data from the National COVID Cohort Collaborative (N3C), a centralized, harmonized, high-granularity electronic health record (EHR) repository to investigate potential associations of NSAID use in a large, multi-center database [29]. We investigated twelve NSAIDs (celecoxib, diclofenac, droxicam, etodolac, ketorolac, ibuprofen, indomethacin, lornoxicam, meloxicam, naproxen, piroxicam, tenoxicam) that were previously evaluated for their effect on COVID-19 severity in a smaller cohort earlier in the COVID-19 pandemic [27]. Our study focused on hospitalized patients with moderate and severe COVID-19. Our results showed significant associations of NSAID use with decreased all-cause mortality, COVID-19 severity, AKI, invasive ventilation, and extracorporeal membrane oxygenation (ECMO). Our analysis confirms and extends analogous findings from previous observational studies to a much larger cohort of patients drawn from 38 distinct centers in a nationally representative database.

## Methods

### Data analysis

Data analysis was performed using the N3C instance of Palantir Foundry (Palantir Technologies Inc., Denver, Colorado). The analysis was structured as a directed acyclic graph of data transformations using the Foundry platform. Individual transformations were implemented using SQL, Python, or R code. Documentation of the source code is included in Additional file 1.

### Design overview, settings, and participants

Patient data were accessed through the N3C (covid.cd2h.org). N3C aggregates and harmonizes EHR data across 66 clinical organizations in the United States, including the Clinical and Translational Science Awards (CTSA) Program hubs. For this study, patient records were extracted from the 38 centers that provided data for all predictors used in the regression analysis described below. Twenty-six centers did not provide values for Body Mass Index (BMI) and were not included in this study. N3C harmonizes EHR data across four clinical data models and provides a unified analytical platform in which data are encoded using the Observational Medical Outcomes Partnership (OMOP) [30] version 5.3.1. N3C also provides shared phenotype definitions such as those for positive COVID-19 laboratory tests and COVID-19 clinical severity categories [29, 31]. Our study analyzed only inpatients, as a preliminary analysis indicated that NSAID use among outpatients was likely to be incompletely captured (Additional file 1: Table S1).

Patients with recorded use of aspirin and acetaminophen, which share some modes of action with NSAIDs, were excluded from the analysis. For each of the twelve NSAIDs and the excluded medications (aspirin and acetaminophen), we constructed a codeset containing concept IDs representing all formulations of the medications using ATLAS (http://atlas-covid19.ohdsi.org/), the graphical user interface designed to construct cohorts and/or concept sets for the OMOP common data model [32]. Concept IDs for topical and ophthalmic NSAID preparations were excluded from these codesets. For each analyzed comorbidity, a codeset was constructed containing concept IDs representing the comorbidity in question. OMOP concept ID codes for all drugs and comorbidities used in this analysis are listed in Supplemental Tables S2 and S3.

Criteria for the current study were determined as follows. The COVID-19 positive cohort was defined as those patients with any encounter after January 1, 2020 and positive SARS-CoV-2 laboratory test (polymerase chain reaction or antigen). For this study, data from up to October 5, 2021 were included. COVID-19 positive patients whose drug era [33] for any of the 12 NSAIDs began on or before the initial date of COVID-19 diagnosis and continued for at least one day after COVID-19 diagnosis were included in the NSAID treated group. As for the 12 NSAIDs, use of aspirin and acetaminophen was defined as a drug era for either of these drugs that began on or before the day of COVID-19 diagnosis and continued for at least one day. All other patients from the COVID-19 positive cohort were used as the control group in propensity matching. For each patient, comorbidities that were diagnosed before the day of diagnosis of COVID-19 were also recorded.

Only patients with complete records (no missing values for any covariate used in propensity matching or logistic regression) were included for further analysis. The most commonly missing data was BMI. Supplemental Figures S1-S2 show similar distributions of age and Charlson Comorbidity Index [34] in the presence or absence of BMI.

### Outcomes

The primary outcome of interest was a COVID-19 clinical severity of “severe” or “mortality/hospice”. Clinical severity was classified into three categories using the Clinical Progression Scale (CPS) established by the World Health Organization (WHO) for COVID-19 clinical research [35]: WHO severity 3); “moderate” (hospitalized without invasive ventilation, WHO severity 4–6); “severe” (hospitalized with invasive ventilation or ECMO, WHO severity 7–9); and “mortality/hospice” (hospital mortality or discharge to hospice, WHO Severity 10) [31]. In our study, severity grade 3 (moderate) was compared against severity grades 4 and 5 (severe or mortality/hospice). For the purposes of our study, the severity grades of mild and mild ED (emergency department) were not included. In effect, this limits our inclusion criteria to inpatients since Mild (WHO severity 1–3) and Mild ED (WHO severity 3) are limited to patients who were not admitted to the hospital [36]. For the logistic regression analysis described below, patients were assigned to COVID-19 severity groups according to the maximum clinical severity during their index encounter [31], which was defined as the medical encounter during which a positive COVID-19 test was documented for the first time. Secondary outcomes were AKI, ECMO, invasive ventilation, and all-cause mortality at any time following COVID-19 diagnosis.

### Study design

#### Statistical analysis

We performed propensity matching using the “nearest” method implemented in the R *MatchIt* package (version 4.1.0). Each patient from the NSAID treated group was matched to the patient in the untreated group with the closest propensity score. The propensity formula included age, race, ethnicity, gender, smoking status, Charlson Comorbidity Index, and BMI, as well as the presence or absence of a diagnosis of the following comorbidities before COVID presentation: alcoholic liver damage, Alzheimer’s disease, cerebral infarction, chronic hepatitis, chronic respiratory disease, dementia associated with another disease, diabetes type 1, diabetes type 2, hepatic failure, hepatic fibrosis, hepatic steatosis, hypertension, hypertensive kidney disease, ischemic heart disease, lupus, malignant neoplasm (lymphoid hematopoietic related tissue), neoplasm, nicotine dependence, nonhypertensive chronic kidney disease, nonischemic heart disease, other liver disease, portal hypertension, psoriasis, rheumatoid arthritis, unspecified dementia, and vascular dementia.

To investigate the association of treatment and other covariates with COVID-19 severity, we performed logistic regression using the *glm* function in R. The dependent variable, COVID-19 severity, was coded as 0 for patients with “moderate” COVID-19 severity and 1 for patients with “severe”, or “mortality/hospice” COVID-19 severity [31]. We assessed the relationship between COVID-19 severity and NSAID use using logistic regression, using as additional predictors age, race, ethnicity, gender, smoking status, Charlson Comorbidity Index, and BMI, as well as the following comorbidities: alcoholic liver damage, Alzheimer’s disease, cerebral infarction, chronic respiratory disease, diabetes type 1, diabetes type 2, hepatic failure, hepatic fibrosis, hypertensive kidney disease, ischemic heart disease, lupus, malignant neoplasm (lymphoid hematopoietic related tissue), neoplasm, nicotine dependence, nonhypertensive chronic kidney disease, nonischemic heart disease, other liver disease, portal hypertension, psoriasis, unspecified dementia, and vascular dementia. For treatment with the medication, we recorded the estimate, the corresponding *p*-value, the odds ratio, and 95% confidence intervals.

We used the *EValue* R package (version 4.1.2) to determine the minimum strength of an unmeasured confounder in the logistic regression that would be required to change the conclusion that the treatment (NSAID use) was associated with the outcome in question. We treated the outcome of increased COVID-19 severity as a non-rare outcome (as it occurred more frequently than 15%), and death, invasive ventilation, AKI, and ECMO as rare outcomes (since the frequency of these outcomes was less than 15%) [37].

### Role of the funding source

The funders had no role in study design, data collection, analysis, interpretation, writing of the report, or in the decision to submit for publication. The corresponding authors had full access to all study data and had final responsibility for the decision to submit for publication.

## Results

**Fig. 1:**
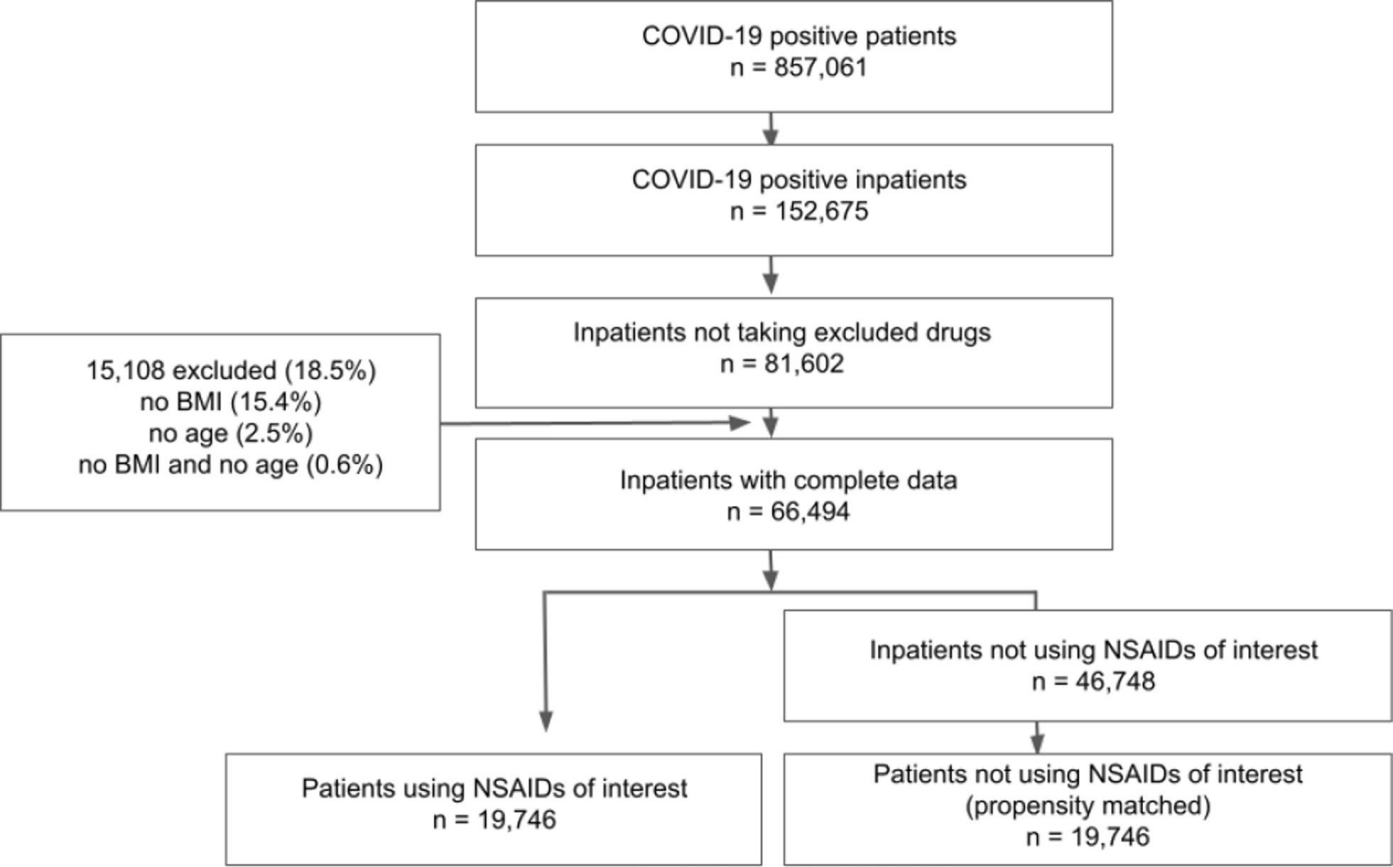
Definition of NSAID cohort and matched control cohort for analysis of the association of NSAID use with COVID-19 outcome

We evaluated 857,061 patients with COVID-19 in a retrospective study and evaluated twelve NSAIDs that were evaluated previously on a smaller cohort [27]. Individuals diagnosed with COVID-19 were then divided into those individuals treated with the medication on the day of admission (treated) and those who were not (controls). To reduce the effect of confounding, we performed propensity matching [38] to match NSAID-treated and control patients according to age, race, ethnicity, gender, smoking status, Charlson Comorbidity Index, BMI, and the presence of 24 comorbidities before COVID-19 presentation (Methods). 66,494 COVID-positive patients had complete data and were not taking aspirin or acetaminophen (excluded drugs), and were included in the analysis. Of these, a total of 19,746 patients were on an NSAID during the 24 h prior to admission and were included in the test cohort, and the same number of patients not on NSAIDs were chosen using propensity matching (Fig. 1). Table 1 shows the composition of the cohort with respect to these covariates before and after propensity matching. After propensity matching, the standard mean difference between NSAID-treated and control groups for all covariates was less than 0.1.

**Table 1:** Characteristics of the COVID-19 positive cohort taking NSAIDs and the control cohort, before and after propensity matching

	Treated	Before propensity matching		After propensity matching	
		Control	SMD	Control	SMD
Age (years)	47.4	53.7	− 0.33	47.1	0.01
Race					
Asian	3.1%	3.7%	− 0.03	3.1%	0.00
Black or African American	21.6%	20.9%	0.02	22.3%	− 0.02
Missing/unknown	25.1%	23.3%	0.04	24.8%	0.01
Native Hawaiian or Other Pacific Islander	0.2%	0.3%	− 0.01	0.2%	0.00
Other	0.9%	0.9%	− 0.01	0.8%	0.01
White	49.1%	50.9%	− 0.04	48.9%	0.00
Ethnicity					
Hispanic or Latino	24.8%	22.0%	0.06	23.8%	0.02
Missing/unknown	5.4%	8.4%	− 0.13	5.4%	0.00
Not Hispanic or Latino	69.8%	69.6%	0.00	70.8%	− 0.02
Gender					
Female	59.7%	50.1%	0.20	59.1%	0.01
Male	40.3%	49.8%	− 0.20	40.9%	− 0.01
Other	0.0%	0.0%	0.01	0.0%	0.00
Smoking status					
Current or former	39.8%	29.9%	0.20	36.7%	0.06
Non smoker	60.2%	70.1%	− 0.20	63.3%	− 0.06
BMI (kg/m^2^)	31.3	29.7	0.18	31.0	0.03
Charlson Comorbidity Index (mean score)	0.98	1.43	− 0.23	0.96	0.01
Alcoholic liver damage	0.6%	1.0%	− 0.05	0.6%	0.00
Chronic hepatitis	1.0%	1.1%	− 0.01	0.9%	0.01
Diabetes type 2	16.7%	18.0%	− 0.03	16.1%	0.02
Hepatic failure	0.5%	1.2%	− 0.11	0.5%	0.00
Hypertension	30.8%	31.3%	− 0.01	29.9%	0.02
Ischemic heart disease	4.9%	7.0%	− 0.10	4.9%	0.00
Lupus	0.7%	0.6%	0.02	0.7%	0.00
Malignant neoplasm (lymphoid hematopoietic related tissue)	2.0%	2.0%	0.00	2.0%	0.00
Neoplasm	19.4%	17.2%	0.06	19.2%	0.00
Nonischemic heart disease	20.5%	24.4%	− 0.10	20.4%	0.00
Vascular dementia	0.4%	0.8%	− 0.07	0.3%	0.00
Alzheimer's disease	0.4%	1.0%	− 0.09	0.4%	0.01
Cerebral infarction	1.7%	2.9%	− 0.10	1.6%	0.01
Chronic respiratory disease	13.1%	13.2%	0.00	12.8%	0.01
Dementia associated with another disease	1.0%	1.5%	− 0.05	0.9%	0.01
Diabetes type 1	1.4%	1.5%	− 0.01	1.4%	0.00
Hepatic fibrosis	1.2%	2.2%	− 0.09	1.2%	0.00
Hepatic steatosis	4.3%	3.2%	0.06	4.0%	0.02
Hypertensive kidney disease	3.1%	6.3%	− 0.19	3.1%	0.00
Nicotine dependence	9.8%	8.9%	0.03	9.6%	0.01
Nonhypertensive chronic kidney disease	5.5%	11.2%	− 0.25	5.4%	0.00
Other liver disease	5.2%	5.4%	− 0.01	4.8%	0.02
Portal hypertension	0.5%	1.2%	− 0.10	0.5%	0.00
Rheumatoid arthritis	1.9%	1.3%	0.04	1.8%	0.01
Unspecified dementia	1.2%	2.7%	− 0.14	1.1%	0.01
Psoriasis	1.0%	0.8%	0.01	0.9%	0.00

The primary outcome was COVID-19 severity of moderate vs. severe or mortality/hospice (Table 2). No mild or mild ED cases were present in our treated or control groups. The incidence of moderate COVID-19 was higher among the NSAID cohort (91.3%) compared with the control cohort (86%), while the incidence of COVID-19 more severe than moderate (severe, or dead) was lower in the NSAID cohort (3.9% severe, 4.8% dead) compared to the control cohort (5.3% severe, 8.8% dead). Similarly, the incidence of invasive ventilation, AKI, and ECMO were all lower in the NSAID group compared to the control group.

**Table 2:** Outcomes in NSAID cohort and propensity-matched control cohort

	NSAID	Control
*COVID-19 severity*		
Moderate	18,023 (91.3%)	16,972 (86%)
Severe	776 (3.9%)	1047 (5.3%)
Dead	947 (4.8%)	1727 (8.8%)
Invasive ventilation	1150 (5.8%)	1867 (9.5%)
AKI	1729 (8.8%)	2437 (12.3%)
ECMO	54 (0.27%)	109 (0.55%)

Number of patients and percent of cohort for each outcome are shown

*AKI* acute kidney injury; *ECMO* extracorporeal membrane oxygenation

We observed a significant association between NSAID use and lower COVID-19 severity when controlling for age, race, ethnicity, gender, smoking status, Charlson comorbidity, BMI, and 22 other comorbidities (OR 0.57, Table 3).

**Table 3:** Association of NSAID use with COVID-19 outcomes as measured by logistic regression. AKI: acute kidney injury; ECMO: extracorporeal membrane oxygenation

	OR (95% CI)	*p* value
COVID severity (severe or dead)	0.57 (0.53–0.61)	< 0.0001
Mortality/hospice	0.51 (0.47–0.56)	< 0.0001
Invasive ventilation	0.59 (0.55–0.64)	< 0.0001
AKI	0.67 (0.63–0.72)	< 0.0001
ECMO	0.51 (0.36–0.7)	< 0.0001

We further investigated the association of NSAID use with four secondary outcomes: AKI, ECMO, invasive ventilation, and all-cause mortality at any time following COVID-19 diagnosis. The characteristics of the NSAID treated and control cohorts with respect to outcomes are shown in Table 2. We analyzed the association of NSAID use with these four other secondary outcomes using logistic regression (Table 3). NSAID use was significantly associated with fewer incidents of death (OR 0.51), invasive ventilation (OR 0.59), AKI (OR 0.67), and ECMO (OR 0.51).

### Quantitative bias analysis

We calculated the E-value for the observed values of the odds ratio to assess the sensitivity of our findings to uncorrected confounders [37]. To fully explain the association of NSAID use with decreased COVID-19 severity, an unmeasured confounder would need to be associated with both the treatment and the outcome with an odds ratio of at least 1.88 above and beyond the confounders included in the regression. Likewise, to fully explain the association of NSAID use with fewer adverse secondary outcomes, a confounder would need to be associated with both the treatment and the outcome with an odds ratio of at least 3.3 (death), 2.8 (invasive ventilation), 2.3 (AKI), and 3.3 (ECMO).

## Discussion

To our knowledge, randomized clinical trial data investigating potential beneficial or deleterious effects of NSAIDs on the course of COVID-19 are not available. Previous observational studies have failed to show an association of exposure to NSAIDS with risk of hospital admission, severe clinical course, or death in COVID-19 patients [39]. Our study is the second-largest to be performed to date and the largest to be performed as a multi-center American study (Supplemental Table S4). The largest study leveraged data in the OpenSAFELY platform, which includes information from primary care practices in England, including pseudonymised data such as coded diagnoses, prescribed medications, and physiological parameters. Our study represents the largest multi-center study with data harmonized from multiple EHR data sources.

Our findings did not show an association in hospitalized COVID-19 patients between NSAID use and increased COVID-19 severity, or increased risk of invasive ventilation, AKI, ECMO, and all-cause mortality. In contrast, we identified a significant association between NSAID use and *decreased* risk of these outcomes. These results are in accordance with those of some previous studies: NSAID use was reported less frequently among hospitalized patients than non-hospitalized patients [16]; a study on 1305 hospitalized COVID-19 patients showed that use of NSAIDs prior to hospitalization was associated with lower odds of mortality as assessed by multivariate regression analysis [25]; finally, the OpenSAFELY study demonstrated a lower risk of COVID-19-related death was associated with current use of NSAIDs in individuals with rheumatoid arthritis/osteoarthritis [28]. However, the E-values for the associations identified by our study were in the range of 1.9–3.3, indicating that comparatively weak or moderate confounder associations could explain away the observed associations. We interpret the results of our study as providing additional evidence for the lack of a detrimental effect of NSAID use prior to hospital admission on the severity and other deleterious outcomes of COVID-19. Future work will be required to investigate a potential beneficial effect.

### Strengths and limitations of this study

Our dataset is derived from over 38 institutions across the country with 857,061 cases of COVID-19, and thus is a representative sample of the COVID-19 positive population in the United States. Observational studies such as retrospective EHR cohort analyses are subject to confounding. In the case of our study, the decision of whether to treat a patient with an NSAID could in principle be correlated with the outcome of interest (COVID-19 severity). We applied propensity matching to mitigate confounding, and used E-values to measure the strength of a confounder that would be required to change the conclusion of our analysis. However, in observational studies a risk of residual confounding persists because the efficacy of propensity matching is limited to known and measured factors. Exposure to the drugs of interest, most of which are available without a prescription, was likely to be captured incompletely in the EHR data used in the analysis. Thus, it is possible that there was unrecorded use of NSAIDs in the untreated group. Our study analyzed only inpatients, whose NSAID use is likely more to be completely captured by EHR data.

## Conclusions

The results of our observational study failed to demonstrate a significant association of the use of twelve NSAIDs with increased clinical severity, invasive ventilation, and AKI. Our study provides additional support to the notion that NSAIDs are safe for use in patients with COVID-19.

## Supplementary Information

**Additional file 1.** Figures S1–S2, Tables S1–S4, and Supplemental Note 1.

## Data Availability

Source code used in this study is available in the supplemental data. Data are available within the N3C Enclave.
